# The Neuroprotective Effects of *Ratanasampil* on Oxidative Stress-Mediated Neuronal Damage in Human Neuronal SH-SY5Y Cells

**DOI:** 10.1155/2015/792342

**Published:** 2015-05-03

**Authors:** Aiqin Zhu, Zhou Wu, Jie Meng, Patrick L. McGeer, Yi Zhu, Hiroshi Nakanishi, Shizheng Wu

**Affiliations:** ^1^Institution of Geriatric Qinghai Provincial Hospital, Xining 810007, China; ^2^Department of Aging Science and Pharmacology, Faculty of Dental Science, Kyushu University, Fukuoka 812-8582, Japan; ^3^Kinsmen Laboratory of Neurological Research, Department of Psychiatry, The University of British Columbia, 2255 Wesbrook Mall, Vancouver, BC, Canada V6T 1W5; ^4^Hospital Infection Management Office, Hospital of Xinjiang Production and Construction Corps, Urumqi, Xinjiang 830002, China

## Abstract

We previously found that *Ratanasampil* (RNSP), a traditional Tibetan medicine, improves the cognitive function of mild-to-moderate AD patients living at high altitude, as well as learning and memory in an AD mouse model (Tg2576); however, mechanism underlying the effects of RNSP is unknown. In the present study, we investigated the effects and molecular mechanisms of RNSP on oxidative stress-induced neuronal toxicity using human neuroblastoma SH-SY5Y cells. Pretreatment with RNSP significantly ameliorated the hydrogen peroxide- (H_2_O_2_-) induced cytotoxicity of SH-SY5Y cells in a dose-dependent manner (up to 60 *μ*g/mL). Furthermore, RNSP significantly reduced the H_2_O_2_-induced upregulation of 8-oxo-2′-deoxyguanosine (8-oxo-dG, the oxidative DNA damage marker) but significantly reversed the expression of repressor element-1 silencing transcription factor (REST) from H_2_O_2_ associated (100 *μ*M) downregulation. Moreover, RNSP significantly attenuated the H_2_O_2_-induced phosphorylation of p38 mitogen-activated protein kinase (MAPK) and extracellular signal-regulated kinase 1/2 (ERK 1/2) in SH-SY5Y cells. These observations strongly suggest that RNSP may protect the oxidative stress-induced neuronal damage that occurs through the properties of various antioxidants and inhibit the activation of MAPKs. We thus provide the principle molecular mechanisms of the effects of RNSP and indicate its role in the prevention and clinical management of AD.

## 1. Introduction

By the year 2030, roughly 20% of the world's population will be over the age of 65 years [1]. As the mean life expectancy continues to increase, the cognitive impairments associated with Alzheimer's disease (AD) will be one of the major issues in aging societies around the world. Oxidative stress is a major component of the harmful cascades activated in the development of aging-related neurodegenerative disorders, including AD [2–4], because oxidative stress through the overproduction of reactive oxygen species (ROS) causes cell damage through the promotion of lipid peroxidation, DNA damage, and the regulation of death proteins [5]. Numerous reports have provided direct morphological and biochemical evidence indicating the connection between oxidative stress and cell death in the brain of AD patients [6–8]. Antioxidant therapy is considered to be one of the promising approaches to the prevention and clinical management of AD [9].

Mitogen-activated protein kinase (MAPK) cascades are sensitive to oxidative stress. The overproduction of ROS during oxidative stress plays a role as a second messenger in signal transduction cascades leading to the activation of MAPKs, because the intracellular redox state of the cells regulates the cellular signaling pathways [10, 11]. The members of the major MAPK subfamily, c-Jun N-terminal kinase (JNK), p38 MAPK, and extracellular signal-regulated kinase 1/2 (ERK 1/2), are known cell death factors that increase in response to oxidative stress [12, 13]. Indeed, increased levels of phosphorylated MAPKs have been found in the postmortem examinations of the brains of AD patients [14–16].

*Ratanasampil* (RNSP), the Tibetan language name which translates to* “seventy-taste pearl-balls,”* has been traditionally used for the treatment of stroke and cerebrovascular diseases. Our previous studies have found that RNSP increases learning and memory and reduces *β*-amyloid (A*β*) protein levels in mouse AD models (Tg2576) [17, 18]. Furthermore, we have recently found that RNSP improves the cognitive functions in mild-to-moderate AD patients living at high altitude and decreases serum A*β*_42_ concentration as well as proinflammatory factors, including Tumor Necrosis Factor (TNF-*α*) and interleukins IL-1*β* and IL-6. These findings indicate the therapeutic effects of RNSP in the prevention and clinical management of AD [19]. However, the mechanism underlying these effects has remained unknown.

RNSP is composed of more than seventy components, including saffron and* Glycyrrhiza uralensis* [20, 21]. The beneficial effects of RNSP components have been received. Previous studies have shown that saffron effectively inhibited the TNF*α*-induced cell death of PC12 cells [22] and protected neurons from the neurotoxic activity of 6-hydroxydopamine hydrobromide [23], via its antioxidant properties [24–26]. More recent studies have reported that* Glycyrrhiza uralensis* exerts anti-inflammatory effects by inhibiting the activation of nuclear factor-kappa B (NF*κ*B) as well as the NOD-like receptor family, a pyrin domain containing 3 (NLRP3) inflammasome [27, 28]. In the present study, we focused on the effects and molecular mechanisms of RNSP in hydrogen peroxide- (H_2_O_2_-) induced neuronal death using cultured SH-SY5Y cells, because H_2_O_2_ is one of the most important ROS generated during oxidative stress [29, 30] and because SH-SY5Y cells are widely used for the study of neuronal cell death induced by oxidative stress [31, 32]. Pretreatment with RNSP was found to significantly ameliorate the H_2_O_2_-induced cytotoxicity of SH-SY5Y cells in a dose-dependent manner (from 10 *μ*g/mL to 60 *μ*g/mL). Furthermore, RNSP was found to significantly reduce the H_2_O_2_-upregulated expression of 8-oxo-deoxyguanosine (8-oxo-G, major product of DNA oxidation that is widely used as an oxidative DNA damage marker) but significantly reserved repressor element-1 silencing transcription factor (REST) from H_2_O_2_-related downregulation. Moreover, RNSP significantly attenuated the H_2_O_2_-induced phosphorylation of p38 and ERK1/2 in SH-SY5Y cells. These observations strongly suggest that RNSP may provide a protective effect against oxidative stress-induced neuronal death via its antioxidant properties and through the inhibition of MAPK activation. The findings of this study may thus provide the principle molecular mechanisms supporting the clinical usage of RNSP in the prevention and clinical management of AD.

## 2. Materials and Methods

### 2.1. Reagents

*Ratanasampil* (RNSP, Zhunzi Z63020062) was purchased from Qinghai Jinke Tibetan Medicine Pharmaceutical Co. Ltd. (Xining, China). A suitable concentration of methanol was titrated for cell culture in order to prevent interference from the methanol solvent. H_2_O_2_ (30%) was purchased from Sigma-Aldrich (St. Louis, MO, USA). Mouse monoclonal anti-8-oxo-deoxyguanosine (8-oxo-dG) was purchased from NOF Corporation (Kyoto, Japan); rabbit anti-phospho-p38, rabbit anti-p38, rabbit anti-phospho-pERK1/2, rabbit anti-phospho-ERK1/2, rabbit anti-phospho-pJNK (1 : 1000), and rabbit anti-pJNK (1 : 1000) were purchased from Cell Signaling Technology (Danvers, MA, USA).

### 2.2. SH-SY5Y Cell Culture

Cells of the SH-SY5Y human neuroblastoma cell line, which were purchased from American Type Culture Collection (Manassas, VA, USA), were cultured in DMEM/F-12 mixture supplemented with 10% fetal bovine serum (FBS, ICN Biomedicals, Eschwege, Germany), 2 mM L-glutamine, and 1% antibiotic and antimycotic solution (Sigma, St. Louis, MO, USA) in a humid atmosphere of 5% CO_2_ and 95% air at 37°C. To determine the toxicity of the reagents, the cells were treated with freshly prepared H_2_O_2_ (from 30% stock) at concentrations ranging from 5 to 500 *μ*M (diluted with sterile purified water), and RNSP was tested at concentrations ranging from 0.01 to 100 ug/mL (diluted with methanol or sterile purified water) for different treatment times.

### 2.3. Cell Survival Assay

An MTT assay was performed as described previously [33]. Based on the preliminary observations, cells in the exponential phase of growth were seeded in 96-well plates (1 × 10^5^ cells/well), allowed to adhere to plates (for 24 h), and treated with various concentrations of RNSP (diluted with methanol or sterile purified water), H_2_O_2_ (diluted with sterile purified water), and vehicle (methanol or sterile purified water). The relative cell viability was measured using CellQuanti-MTT Cell Viability Assay Kits (Bio Assay Systems, Hayward, USA). The assay was performed according to the manufacturer's protocol. The absorbency at 570 nm was evaluated using a microplate reader.

### 2.4. Observations of Morphological Changes

The cells were seeded in 24-well plates (2 × 10^5^ cells/well) for 24 h and then treated with H_2_O_2_ at a concentration of 100 *μ*M for 24 h with or without RNSP (methanol extraction) at different concentrations. The cellular morphology was observed and photographed using a bright-field microscope (Nikon, ECLIPSE Ti-S, Japan).

### 2.5. Detection of Mitochondrial ROS

Mitochondrial ROS were measured using MitoSOX Red (Invitrogen, USA), which is a live-cell permeant that rapidly and selectively targets mitochondria [33]. Once in the mitochondria, MitoSOX Red reagent is oxidized by superoxide and exhibits red fluorescence (with excitation at 510 nm and emission at 580 nm). The cultured SH-SY5Y cells were seeded in 24-well plates (2 × 10^5^ cells/well) and incubated with or without RNSP for 2 h (methanol extraction, 60 *μ*g/mL). The cells were then further exposed to H_2_O_2_ (100 *μ*M) for 1 h. After incubation in Hank's balanced salt solution (HBSS) containing 5 mM MitoSOX Red for 10 min at 37°C, the cells were washed twice with PBS and then mounted in a warm buffer for imaging. Images were collected using a ×20 objective lens (NA = 0.50, 200x magnification, yielding a frame of 0.575 mm^2^). The procedure resulted in arbitrary optical density values on a scale of 0 (background staining) to 255.

### 2.6. Real-Time Quantitative RT-PCR Analysis

SH-SY5Y cells were seeded in a 10 cm^2^ dish (1 × 10^7^/dish), which was pretreated with RNSP (methanol extraction, 60 *μ*g/mL) for 2 h. The cells were then treated with H_2_O_2_ (100 *μ*M) for 1 or 2 h. The mRNAs isolated from the RNSP-pretreated or nontreated cells were subjected to a real-time quantitative RT-PCR. The total RNA was extracted with a PureLink RNA micro kit (Invitrogen, Japan), which was used in accordance with the manufacturer's instructions. A total of 800 ng of extracted RNA were reverse-transcribed to cDNA using High Capacity RNA-to-cDNA Master Mix (Applied Biosystems, Foster City, CA). Thermal cycling was conducted at 50°C for 2 min and then at 95°C for 10 min, followed by 40 cycles of 95°C for 15 s and 60°C for 1 min. The cDNA was amplified in duplicate using TaqMan Universal PCR Master Mix (Applied Biosystems, Foster City, CA) with an Applied Biosystems 7500/7500 Fast Real-Time PCR System. The data were evaluated using the 7500 software program (version 2.0, Applied Biosystems). The following primer sequences were used: repressor element-1 silencing transcription factor (REST): 5′-TGG TGG GTG CCC AAA TTG TA-3′ and 5′-ACC TGC ATG GGA GCA GAT TC-3′. For data normalization, an endogenous control (actin) was assessed to control for the cDNA input, and the relative units were calculated using a comparative Ct method. All of the real-time RT-PCR experiments were repeated three times, and the results are presented as the means of the ratios ± SEM.

### 2.7. Immunofluorescence Imaging

Immunofluorescence imaging was performed as described previously [34]. Briefly, SH-SY5Y cells were seeded in 24-well plates (2 × 10^5^ cells/well) for 24 h, then treated with H_2_O_2_ (100 *μ*M) for 4 h, with or without pretreatment with RNSP (methanol extraction, 60 *μ*g/mL) for 2 h, and fixed with 4% paraformaldehyde. After washing the cells with PBS twice, they were incubated with mouse anti-8-oxo-dG (1 : 500) overnight at 4°C and then incubated with anti-mouse Alexa 488 (1 : 2000, Jackson ImmunoResearch Lab. Inc.) at 4°C for 2 h. The cells were mounted in the antifading medium, VECTASHIELD, and the fluorescence images were taken using a confocal laser scanning microscope (CLSM, C2si, Nikon, Japan).

### 2.8. Electrophoresis and Immunoblotting

SH-SY5Y cells were cultured at a density of 1 × 10^7^ cells in 10 cm^2^ dishes. The cytosolic samples of the cells were collected at 10, 30, and 60 min after H_2_O_2_ (100 *μ*M) treatment, with or without RNSP (methanol extraction, 60 *μ*g/mL). The lysed samples were electrophoresed in 12% SDS-polyacrylamide gels, and the proteins on the SDS gels were electrophoretically transferred to nitrocellulose membranes. Following blocking, the membranes were incubated at 4°C overnight under gentle agitation with each primary antibody: rabbit anti-phospho-p38 (1 : 1000), rabbit anti-p38 (1 : 1000), rabbit anti-phospho-pERK1/2 (1 : 1000), rabbit anti-phospho-ERK1/2 (1 : 1000), rabbit anti-phospho-pJNK (1 : 1000), rabbit anti-pJNK (1 : 1000), and rabbit anti-phospho-pERK1/2 (1 : 1000) antibodies. After washing, the membranes were incubated with horseradish peroxidase- (HRP-) labeled anti-rabbit (1 : 2000, GE Healthcare, UK) antibody for 2 h at 24°C. The protein bands were then detected using an enhanced chemiluminescence detection system (ECK kit, Amersham Pharmacia Biotech) with an image analyzer (LAS-4000, Fuji Photo Film, Tokyo, Japan).

### 2.9. Statistical Analysis

The data are represented as the means ± SEM. Statistical analyses were performed using one-way or two-way analysis of variance (ANOVA) with post hoc Tukey's test using the GraphPad Prism software package. A *p* value of <0.05 was considered to indicate statistical significance (GraphPad Software Inc., San Diego, CA, USA).

## 3. Results

### 3.1. Effect of RNSP on Cell Viability in SH-SY5Y Cells

**Figure 1:**
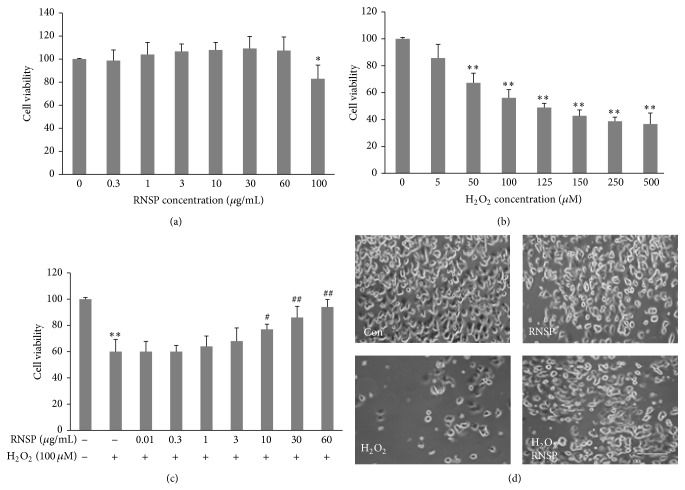
The effects of RNSP on H_2_O_2_-induced toxicity in SH-SY5Y cells. (a) The cell viability of SH-SY5Y cells after treatment with RNSP for 5 days. (b) The cell viability of SH-SY5Y cells after 24 h of exposure to H_2_O_2_. (c) The effect of RNSP on H_2_O_2_-induced toxicity in SH-SY5Y cells. Pretreatment was performed with RNSP (5 days) before exposure to H_2_O_2_ (100 *μ*M, 24 h). Each column and bar represent the mean ± SEM (*n* = 4 each). Asterisks indicate a statistically significant difference from the value in untreated cells (^∗^ *p* < 0.05, ^∗∗^ *p* < 0.01). Pound signs indicate a statistically significant difference from the value in H_2_O_2_-exposed cells without pretreatment with RNSP (^#^ *p* < 0.05, ^##^ *p* < 0.01). (d) The morphological changes of SH-SY5Y cells with or without pretreatment with RNSP (60 *μ*g/mL, 5 days) after exposure to H_2_O_2_ for 24 h. Scale bar = 20 *μ*m.

We first examined the effects of RNSP on the cell viability of SH-SY5Y cells using an MTT assay. The mean cell viability was not significantly changed after treatment with the methanol extracts of RNSP at final concentrations between 0.3 and 60 *μ*g/mL for 5 days (Figure 1(a)). However, the mean cell viability was significantly reduced after pretreatment with RNSP at a final concentration of over 100 *μ*g/mL (82% of viable cells). It is known that the concentration of H_2_O_2_ in healthy individuals is normally quite low, and the elevation of H_2_O_2_ concentration upon cerebral ischemic and reperfusion injury could act as a significant signal [35, 36]. We next examined the toxic effect of H_2_O_2_ at different concentrations (5–500 *μ*M) in SH-SY5Y cells, because H_2_O_2_ has frequently been used as an oxidative stimulus to identify redox-sensitive processes. The viability of SH-SY5Y cells was decreased after exposure to H_2_O_2_ for 24 h in a dose-dependent manner. In comparison to nonexposed cells, 56.8% of the viable cells were detected at an H_2_O_2_ concentration of 100 *μ*M, while less than 50% of the viable cells were detected at an H_2_O_2_ concentration of over 125 *μ*M (Figure 1(b)). We therefore used methanol extracts of RNSP at concentrations of up to 60 *μ*g/mL and H_2_O_2_ at 100 *μ*M concentration to investigate the effects of RNSP on the H_2_O_2_-induced cytotoxicity of SH-SY5Y cells in the subsequent experiments.

### 3.2. The Effects of RNSP on H_2_O_2_-Induced Toxicity in SH-SY5Y Cells

**Figure 2:**
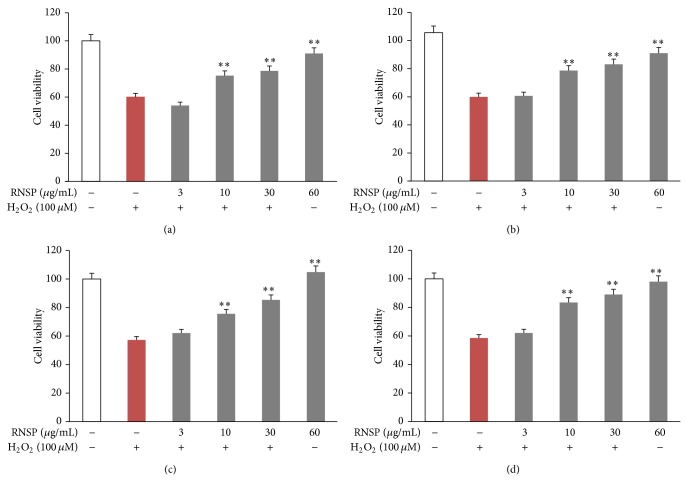
The effects of RNSP in different solvents and times on H_2_O_2_-induced toxicity in SH-SY5Y cells. Pretreatment with water extracts (a) and methanol extracts (b) of RNSP for 5 days. Each column and bar represent the mean ± SEM (*n* = 4 each). Asterisks indicate a statistically significant difference from the value in the H_2_O_2_-exposed cells without RNSP (^∗∗^ *p* < 0.01). The effect of pretreatment with methanol extracts of RNSP for 2 h (c) and 2 h two times in a 2 h interval (2-2 h) (d) on H_2_O_2_-exposed SH-SY5Y cells. Each column and bar represent the mean ± SEM (*n* = 4 each). Asterisks indicate a statistically significant difference from the value in the H_2_O_2_-incubated cells without RNSP (^∗∗^ *p* < 0.01).

Pretreatment with methanol extracts of RNSP for 5 days prevented H_2_O_2_-induced cytotoxicity in SH-SY5Y cells in a dose-dependent manner (Figure 1(c)). In comparison to the H_2_O_2_-treated cells, the cell viabilities were significantly restored from treatment with RNSP at a concentration of 10 *μ*g/mL, and 92.8% of viable cells were detected in RNSP with a concentration of 60 *μ*g/mL. The protective effect of RNSP was further confirmed by the microscopic evaluation of the cells. Morphological changes that were observed included the degeneration of H_2_O_2_-treated SH-SY5Y cells, which exhibited the disappearance of the neuritis and shrinkage (Figure 1(d)). The percentage of viable cells was also reduced by H_2_O_2_ in a dose-dependent manner (data not shown). It is noted that the neuritis and shrinkage of cells were attenuated by pretreatment with RNSP (Figure 1(d)). To determine the effects of the soluble components of RNSP, we further examined the effects of water extracts of RNSP on H_2_O_2_-induced toxicity in SH-SY5Y cells in comparison to the methanol extracts of RNSP. As shown in Figures 2(a) and 2(b), pretreatment with the RNSP water extracts for 5 days reduced the H_2_O_2_-induced toxicity in SH-SY5Y cells in a dose-dependent manner. No significant difference was detected between the water and methanol extracts (Figures 2(a) and 2(b)). To further address the protective effects of RNSP over short time periods, pretreatment with methanol extracts of RNSP for 2 h as well as twice for 2 h with a 2 h interval (2-2 h) was performed in SH-SY5Y cells before exposure to H_2_O_2_ (24 h). As shown in Figures 2(c) and 2(d), pretreatment with RNSP for both 2 h and 2-2 h significantly inhibited the H_2_O_2_-induced cytotoxicity in SH-SY5Y cells in a dose-dependent manner, and no significant difference was detected between the two treatment schedules in the effects on the H_2_O_2_-induced toxicity in cells. Taken together, these observations strongly demonstrate that pretreatment with RNSP protects SH-SY5Y cells from H_2_O_2_-induced cytotoxicity. Pretreatment with methanol extracts of RNSP (60 *μ*g/mL) for 2 h was set up in the subsequent experiments.

### 3.3. The Effects of RNSP on H_2_O_2_-Induced DNA Damage in SH-SY5Y Cells

**Figure 3:**
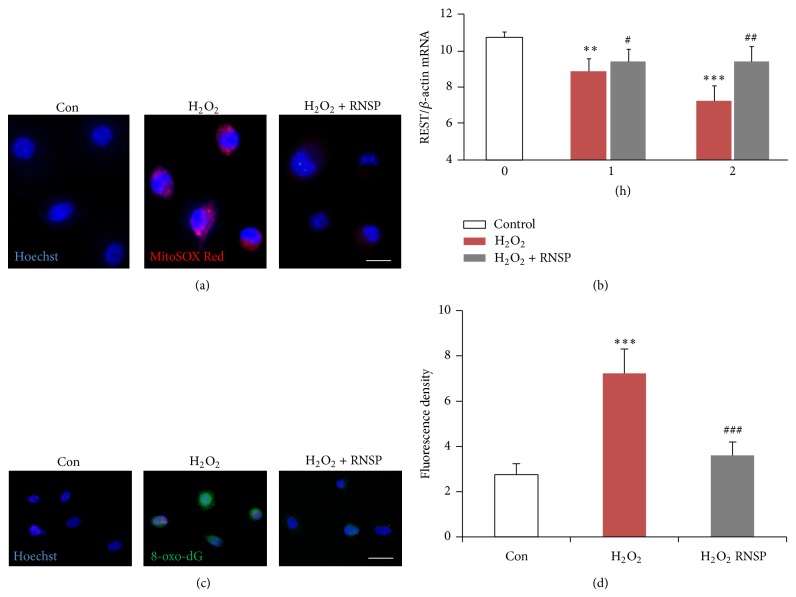
The effects of RNSP on H_2_O_2_-induced DNA damage in SH-SY5Y cells. (a) Fluorescent images of MitoSOX Red signals in the H_2_O_2_-incubated cells (100 *μ*M, 1 h) with or without RNSP (60 *μ*g/mL, 2 h). Scale bar = 10 *μ*m. (b) REST expression, as analyzed by real-time quantitative RT-PCR, in the H_2_O_2_-exposed cells (100 *μ*M, 1 h, 2 h), with or without RNSP (60 *μ*g/mL, 2 h). Each column and bar represent the mean ± SEM (*n* = 4 each). Asterisks indicate a statistically significant difference from the value in the untreated cells (^∗∗∗^ *p* < 0.01, ^∗∗^ *p* < 0.001). Pound signs indicate a statistically significant difference from the value in the H_2_O_2_-incubated cells without RNSP (^#^ *p* < 0.05, ^##^ *p* < 0.01). (c) Immunofluorescent CLMS images of 8-oxo-dG (green) with Hoechst-stained nuclei (blue) in the H_2_O_2_-exposed cells (100 *μ*M 4 h), with or without RNSP (60 *μ*g/mL, 2 h). Scale bar = 10 *μ*m. (d) The quantitative analyses of 8-oxo-dG immunofluorescence signal intensity in (c). Each column and bar represent the mean ± SEM (*n* = 4 each). Asterisks indicate a statistically significant difference from the value in the untreated cells (^∗∗∗^ *p* < 0.001). Pound signs indicate a statistically significant difference from the value in the H_2_O_2_-exposed cells without RNSP (^###^ *p* < 0.001).

Oxidative stress is an important inducer of neurotoxicity in AD patients [37], causing damage to cardinal cellular components, including the DNA, and initiating subsequent cell death [38]. Following our previous experiments, we used two approaches to address the effects of RNSP on H_2_O_2_-induced DNA damage in SH-SY5Y cells: one approach was the use of a MitoSOX Red probe, as a marker for mitochondria-derived ROS generation [33], and the other was immunofluorescence imaging for a biomarker of oxidation-damaged DNA marker, 8-oxo-dG [39]. In comparison to the untreated cells, the expression of MitoSOX Red signals was significantly increased in SH-SY5Y cells after exposure to H_2_O_2_ for 1 h, suggesting that the mitochondria are the early origin of ROS generation during oxidative stress. Pretreatment with RNSP significantly inhibited the H_2_O_2_-induced mitochondria-derived ROS generation in SH-SY5Y cells (Figure 3(a)), thus confirming the antioxidant properties of RNSP. Immunofluorescence imaging showed a significant inverse relationship between Hoechst and 8-oxo-dG after exposure of SH-SY5Y cells to H_2_O_2_ for 4 h (Figure 3(c)), and the mean fluorescent intensity of 8-oxo-dG was found to significantly increase in comparison to that in the cells that were not exposed to H_2_O_2_ (7.22 versus 2.75, ^∗∗∗^ *p* < 0.001). It is noted that pretreatment with the RNSP methanol extracts for 2 h significantly reduced the immunofluorescence intensity of 8-oxo-dG in the H_2_O_2_-exposed SH-SY5Y cells (3.60 versus 7.22, ^###^ *p* < 0.001, Figures 3(c) and 3(d)). These observations demonstrate that RNSP could attenuate oxidative stress-induced DNA damage in neuronal cells.

### 3.4. The Effects of RNSP on the H_2_O_2_-Induced Downregulation of REST in SH-SY5Y Cells

Recently, the neuroprotective role of repressor element-1 silencing transcription factor (REST) has been shown in the repression of ROS production and oxidative stress [40]. We thus examined the effects of RNSP on H_2_O_2_-induced REST expression in SH-SY5Y cells. A real-time quantitative RT-PCR analysis showed that the REST expression was significantly reduced after the exposure of SH-SY5Y cells to 100 *μ*M of H_2_O_2_ (reduced to 85% at 1 h and 68% at 2 h in comparison to the nonexposed cells, Figure 3(b)), indicating that the downregulation of REST expression is preceded to H_2_O_2_-induced oxidative DNA damage. To our surprise, pretreatment with methanol extracts of RNSP for 2 h significantly reversed the H_2_O_2_-reduced the expression of REST from 1 h (reversed 87.8% at 1 h and 87.8% at 2 h in comparison with H_2_O_2_-exposed cells, Figure 3(b)). This observation suggests that the reversion of RNSP in H_2_O_2_-reduced REST expression may be involved in preventing oxidative DNA damage in SH-SY5Y cells.

### 3.5. The Effects of RNSP on H_2_O_2_-Mediated MAPK Activation in SH-SY5Y Cells

**Figure 4:**
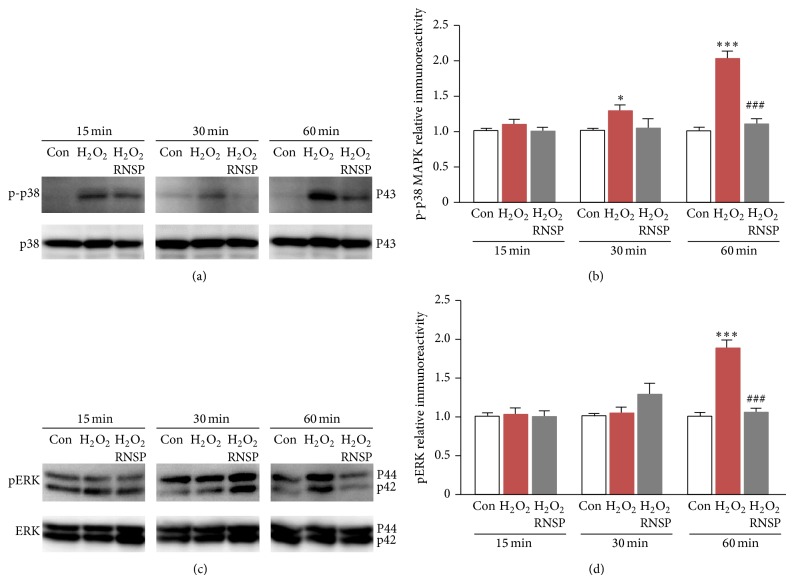
The effects of RNSP on H_2_O_2_-medicated MAPKs activation in SH-SY5Y cells. (a) The phosphorylation of p38 in H_2_O_2_-exposed cells with or without RNSP (60 *μ*g/mL, 2 h). (b) The quantitative analyses of the immunoblotting from (a). Each column and bar represent the mean ± SEM (*n* = 4, each). An asterisk indicates a statistically significant difference from the value in the untreated cells (^∗^ *p* < 0.05, ^∗∗∗^ *p* < 0.001). Pound signs indicate a statistically significant difference from the value in H_2_O_2_-exposed cells without RNSP (^###^ *p* < 0.001). (c) The phosphorylation of pERK1/2 in H_2_O_2_-exposed cells with or without RNSP (60 *μ*g/mL, 2 h). (d) The quantitative analyses of the immunoblotting in (c). Each column and bar represent the mean ± SEM (*n* = 4 each). Asterisks indicate a statistically significant difference from the value in untreated cells (^∗∗∗^ *p* < 0.001). Pound signs indicate a statistically significant difference from the value in the H_2_O_2_-exposed cells without RNSP (^###^ *p* < 0.001).

We finally examined the effects of RNSP on H_2_O_2_-induced p38, ERK1/2, and JNK phosphorylation in SH-SY5Y cells, because they are known to be cell death factors which are mediated by oxidative stress [12, 13]. In comparison to the cells that were not exposed to H_2_O_2_, the mean level of phosphorylated p38 MAPK was significantly increased in SH-SY5Y cells from 30 min to 60 min after exposure to H_2_O_2_ (100 *μ*M) (Figures 4(a) and 4(b)). Along the same lines, the phosphorylated ERK 1/2 MAPK was significantly increased from 60 min after exposure to H_2_O_2_ (Figures 4(c) and 4(d)). However, the mean level of phosphorylated JNK was not significantly increased in SH-SY5Y cells after exposure to H_2_O_2_ (data not shown). Interestingly, pretreatment with the methanol extracts of RNSP for 2 h significantly inhibited the H_2_O_2_-induced phosphorylation of both p38 and ERK 1/2 MAPK from 60 min after incubation with H_2_O_2_ in SH-SY5Y cells (Figures 4(a)–4(d)). However, pretreatment with RNSP for 2 h did not significantly inhibit the phosphorylation of JNK in the SH-SY5Y cells. These observations demonstrate that RNSP could inhibit oxidative stress-induced MAPK activation in SH-SY5Y cells.

## 4. Discussion

**Figure 5:**
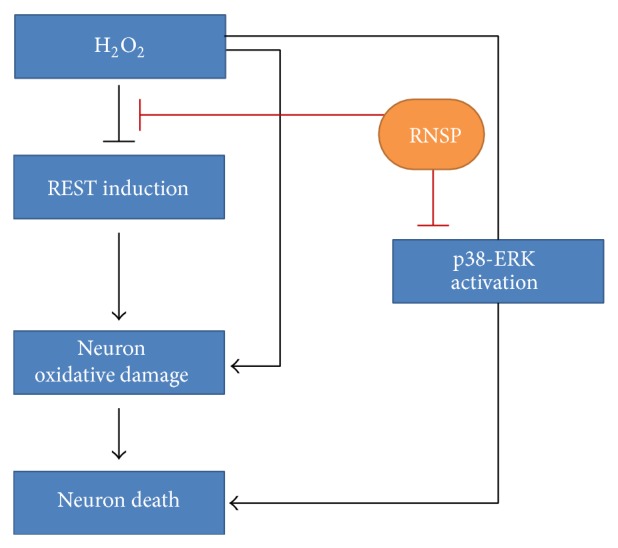
A schematic representation of the effects and the principle molecular mechanisms of RNSP on the protection from oxidative stress-induced neuronal death via the antioxidant properties and inhibition of MAPK activation.

The major findings of the present study are that RNSP protects SH-SY5Y cells from H_2_O_2_-induced neuronal cytotoxicity by reducing the oxidative stress-induced DNA damage and activation of MAPK (summarized in Figure 5). It is known that oxidative stress resulting from the overproduction of ROS causes damage to the cellular components, including DNA, resulting in subsequent cell death [5]. H_2_O_2_ is one of the most important ROS generated through oxidative stress [29, 30]. In the present study, the mean fluorescent intensity of 8-oxo-dG, a biomarker for oxidative stress-damaged DNA [41], was found to be significantly increased, mainly in the nuclei of SH-SY5Y cells from 4 h after exposure to H_2_O_2_. However, the expression of MitoSOX Red probe, a marker for mitochondria-derived ROS generation in the SH-SY5Y cells, increased from 1 h after exposure to H_2_O_2_, thus indicating mitochondrial damage to be the early origin of ROS generation before the nuclear DNA damage that is caused by oxidative stress. It is noted that both the long (5 days) and the short (2 h) pretreatments with RNSP could significantly inhibit the H_2_O_2_-induced mitochondrial ROS generation and nuclear DNA damage (Figures 3(a) and 3(b)) and the subsequent cell death in SH-SY5Y cells (Figures 1 and 2). This is the first evidence support the attenuation of stress-induced DNA damage in neuronal cells by RNSP.

Recent research has shown that REST is related to the reduction of oxidative damage. This was demonstrated by increased levels of ROS as well as oxidative DNA damage in response to REST knockdown. The overexpression of REST (up to 20-fold) can reduce H_2_O_2_-induced neuronal cell death [40]. In the present study, the expression of REST was significantly decreased as little as 1 h after cells were exposed to H_2_O_2_ (100 *μ*M), and the time-dependent decrease of REST expression was positively correlated with subsequent DNA damage. These results agree with the hypothesis that REST knockdown increases oxidative DNA damage [40]. Surprisingly, pretreatment with RNSP for 2 h significantly reversed the H_2_O_2_-induced decrease of REST expression from 1 h (Figure 3(a)), inducing a parallel protective effect against subsequent oxidative DNA damage in SH-SY5Y cells (Figures 3(a) and 3(b)). Since REST is a protective factor in the ageing brain, which is in a state of decline from the early stages of AD [40], and because we have recently found that RNSP taken orally for 16 weeks can significantly improve cognitive function in the early stages of AD [18], the regulation of the H_2_O_2_-mediated REST/oxidative stress may be one of the principle molecular mechanisms behind the protective effects of RNSP that are observed in neuronal cells.

Oxidative stress is known to be one of the major stimulators of the MAPK cascade. The overproduction of ROS during oxidative stress acts as a second messenger in signal transduction cascades leading to MAPK activation, because the intracellular redox state of the cells regulates the cellular signaling pathways [10, 11]. In the present study, p38 and ERK 1/2 MAPKs were significantly activated in SH-SY5Y cells after exposure to H_2_O_2_ (100 *μ*M). This agrees with the hypothesis that ERK and p38 proteins are rapidly activated by H_2_O_2_ in PC12 cells [41, 42]. Pretreatment with RNSP for 2 h significantly reduced the H_2_O_2_-induced phosphorylation of both p38 and ERK1/2, thus indicating that the reduction of the oxidative stress-mediated activation of p38 and ERK 1/2 may be another important molecular mechanism underlying the protective effects of RNSP on neuronal cells, because both p38 and ERK 1/2 MAPK are cell death factors which are mediated by oxidative stress [12, 13].

RNSP is composed of more than seventy components, including saffron and* Glycyrrhiza uralensis* [16, 17], and the beneficial effects of RNSP components have been paid attention [25]. Previous studies have shown that saffron, one of the components of RNSP, inhibits the TNF*α*-induced cell death of PC12 cells [22] and protects neurons from 6-hydroxydopamine hydrobromide-induced neurotoxic activity [23], through antioxidant properties of saffron [24–26]. Our previous studies have found improvements of the cognitive functions in mild-to-moderate AD patients living at high altitude and increases in learning and memory in a mouse model of AD (Tg2576) [17–19]; the present observations have demonstrated the direct roles of RNSP in inhibiting oxidative stress-induced DNA damage and in the activation of MAPKs in neuronal cells, further confirming the benefits of RNSP in the prevention and clinical management of AD [7, 15]. Since oxidative stress is a potential contributor to the pathogenesis of AD [4, 7, 8] and since the levels of phosphorylated MAPKs are increased in the postmortem brains of AD patients [14–16], the antioxidants effects of RNSP can be expected to delay the development of AD.

## 5. Conclusion

RNSP was able to protect oxidative stress-induced neuronal damage via antioxidant properties and the inhibition of MAPK activation (schematic represented in Figure 5). These findings therefore provide the principle molecular mechanisms of the clinical effects of RNSP and support its use as therapeutic agent for the prevention and clinical management of AD.
